# Teaching Hope, Agency, and Possibilities in an Era of Radical Restructuring

**DOI:** 10.1093/geroni/igaf122.398

**Published:** 2025-12-31

**Authors:** Angela Perone

**Affiliations:** University of California, Berkeley, Berkeley, California, United States

## Abstract

The current political, social, and economic moment has brought many new challenges for instructors, students, and the communities we serve. Emerging research suggests parallels with the COVID-19 pandemic and prior historical moments of government retrenchment and overreach. Mental health and economic needs are growing--as are health disparities that have continued to plague the United States and beyond. Higher education is being transformed before our very eyes. And not necessarily always for the better. Despite all these challenges, we as instructors have opportunities to teach our students how to navigate this difficult moment and give ourselves grace to practice (and sometimes fail) in this endeavor. This lecture will provide an overview of case examples, lessons learned, and potential models for teaching hope, agency, and possibilities to support students, colleagues, and ourselves amidst a radical restructuring in higher education and many social, health, and political systems that we study and teach about to our students.

